# Experimental and Computational Analysis of MnO_2_@V_2_C-MXene for Enhanced Energy Storage

**DOI:** 10.3390/nano11071707

**Published:** 2021-06-29

**Authors:** Mahjabeen Fatima, Syedah Afsheen Zahra, Saleem Ayaz Khan, Deji Akinwande, Jan Minár, Syed Rizwan

**Affiliations:** 1 Physics Characterization and Simulations Lab (PCSL), Department of Physics, School of Natural Sciences (SNS), National University of Sciences and Technology (NUST), Islamabad 44000, Pakistan; mahjabeen.fatima96@gmail.com (M.F.); afsheenzahra@outlook.com (S.A.Z.); 2 New Technologies Research Centre, University of West Bohemia, Univerzitni 2732, 306 14 Pilsen, Czech Republic; jmminar@gmail.com; 3 Microelectronics Research Centre, The University of Texas at Austin, Austin, TX 78758, USA; deji@ece.utexas.edu

**Keywords:** V_2_C MXene, energy storage, supercapacitors, two-dimensional materials, density functional theory

## Abstract

Herein, we studied the novel and emerging group of 2D materials namely MXene along with its nanocomposites. This work entails detailed experimental as well as computational study of the electrochemical behavior of vanadium carbide (V_2_CT*_x_*) MXene and MnO_2_-V_2_C nanocomposite with varying percentages of MnO_2_. A specific capacitance of 551.8 F/g was achieved for MnO_2_-V_2_C nanocomposite in 1 M KOH electrolyte solution, which is more than two times higher than the gravimetric capacitance of 196.5 F/g obtained for V_2_C. The cyclic stability achieved for the MnO_2_-V_2_C nanocomposite resulted in a retentivity of 96.5% until 5000 cycles. The c-lattice parameter achieved for MXene is 22.6 Å, which was 13.01 Å for MAX phase. The nanocomposite resulted in a c-lattice parameter of 27.2 Å, which showed that the spatial distance between the MXene layers was efficiently obtained. The method of wet etching was used for the preparation of pristine MXene and the liquid phase precipitation method was opted for the synthesis of the MnO_2_-V_2_C nanocomposite. Density functional theory calculation was exercised so as to complement the experimental results and to understand the microscopic details, such as structure stability and electronic structure. The current report presents a comprehensive experimental and computational study on 2D MXenes for future energy storage applications.

## 1. Introduction

Two-dimensional (2D) materials involve uncommon and rare electronic, mechanical as well as optical properties [1,2,3,4,5], that have led to its wide-ranging analysis for versatile applications in the past decade. In addition, they aid to an expedient building block for diverse layered structures, membranes and composites [6,7,8,9,10]. MXenes—the fresh and newest accoutrement to the world of 2D materials—are basically early transition metal carbides, nitrides and carbonitrides [11,12,13,14,15]. Generally, the formula for MXenes is M*_n_*_+1_X*_n_*T*_x_* (*n* = 1 to 3), whereas M symbolizes an earlier transition metal (Ti, Nb, Ta, Mo, V), X is carbon and/or nitrogen and T*_x_* denotes the surface terminations (hydroxyl, oxygen or fluorine) [16]. Examples may include V_2_CT*_x_* [17], Ti_3_C_2_T*_x_* [18] and Nb_4_C_3_T*_x_* [15]. MXenes involve (*n* + 1) M layers that are enfolded ‘n’ layers of X in an [MX]*_n_*M sequence. MXenes are obtained from selective etching of ‘A’ layers from their lamellar precursors, known as MAX. MXenes are a huge class of ternary carbides and nitrides, including more than 70 reported phases until now, along with various solid solutions and ordered double transition metal structures [19,20,21,22,23,24,25].

Vahid et al. studied electrical and optical properties of two-dimensional V_2_C, revealing intriguing possibilities and opening the door for versatile energy storage applications [26]. Yoon et al. reported delaminated V_2_C MXene with phosphorus obtained from triphenylphosphine intercalated in sheets, for activation of hydrogen evolution reactions from a non-metallic electron donor [27]. Shan et al. recently reported V_2_C MXene as an efficient electrode in various aqueous electrolytes for supercapacitor applications, revealing a different energy density and the highest power density of the V_2_C electrode in several electrolytes [28]. The theoretical insights of V_2_C have also been studied, which reveal it is a capable anode material for lithium-ion batteries and shows a theoretical storage capacity of 940 mAh/g [29,30,31,32].

This article elaborates on the experimental and computational details and insights of pristine V_2_C and the MnO_2_-V_2_C nanocomposite. It observes the trends of vanadium- based MXene when MnO_2_ is adsorbed on the pristine MXene and analyzes the density of states and bandgap via density functional theory [33,34,35], for suitability in energy storage applications.

## 2. Experimental Methods

**Figure 1:**
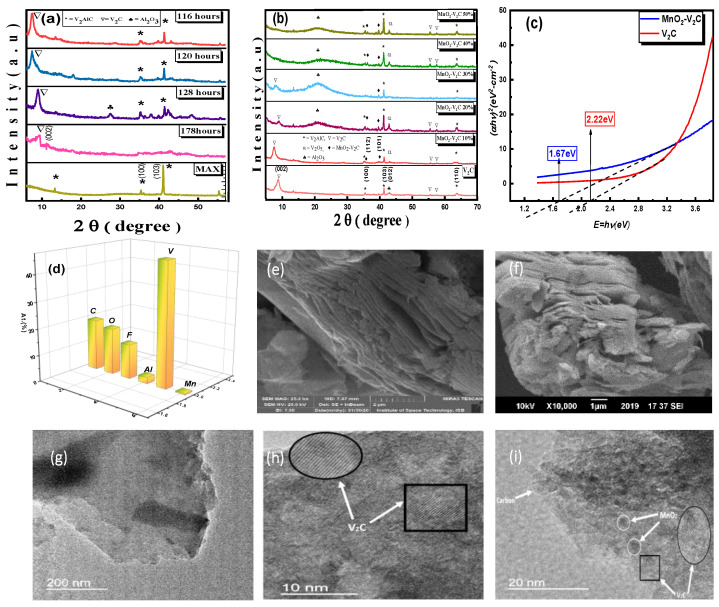
(**a**) XRD of pristine V_2_AlC and prepared V_2_CT*_x_* etched for varying time periods, (**b**) XRD of V_2_C MXene nanocomposites with varying weight percentages of MnO_2_, (**c**) bandgap analysis of V_2_C and MnO_2_-V_2_C nanocomposite, (**d**) EDX for the elemental analysis of MnO_2_-V_2_C nanocomposite, (**e**) micrograph for pristine V_2_C, (**f**) micrograph for MnO_2_-V_2_C nanocomposite. (**g**) TEM image of V_2_C at 200 nm, (**h**) TEM image of V_2_C at 10 nm, (**i**) TEM image of MnO_2_-V_2_C nanocomposite.

Figure 1a shows an XRD of selective etching of Al layer from V_2_AlC to obtain V_2_CT*_x_*. One gram of V_2_AlC MAX (300 mesh) was treated with 49% concentrated hydrofluoric acid for various time periods at room-temperature from which wet-etching for 116 h showed favorable results. The etched sample was washed via centrifugation and vacuum filtration with a powdered sample of V_2_C. However, Pristine V_2_C cannot exist due to its high surface reactivity and readily oxidizes at ambient temperatures. Therefore, MXene with surface terminations was obtained after drying in a vacuum oven for 24 h. The MnO_2_-V_2_CT*_x_* nanocomposite was synthesized by the liquid-phase precipitation method. Two hundred milligrams of V_2_CT*_x_* powder was dispersed in 100 mL of a 1 mM aqueous solution of MnO_2_ with constant magnetic stirring at 40 °C for a time period of 6 h. A precipitate was collected by centrifugation and rinsing with ethanol and DI water separately three times with the help of vacuum filtration. The powder obtained was then dried out in the vacuum oven at 55 °C for 24 h. With this method, five different samples of varying percentages, namely 10%, 20%, 30%, 40% and 50%, were synthesized as the amount of MnO_2_ was increased.

## 3. Results and Discussion

The XRD patterns of the MAX phase and MXene etched with 49% HF solution for different times and divulging structural evolution are shown. The sharp peaks at 2Ө = 13.28°, 41.09° and 55.7° of the MAX precursors represents its high crystallinity and purity. Exposure to HF results in shifting of the (002) peak in the XRD pattern of V_2_AlC to a lower angle, indicating the increased interlayer spacing of synthesized V_2_CT*_x_* MXene (JCPD 03-065-2628). The sample etched for 116 h shows the strongest intensity and the lowest angle of (002) diffraction peaks at 2Ө = 7.4°. The shifting of the peak is the result of an increased c-lattice that is 22.6 Å instead of 13.01 Å for pristine MAX. Small peaks of MAX phase in MXene represent the unetched MXene as suggested by previous studies [36,37,38,39]. The peak at 55.7° persists in MXene due to the presence of traces of MAX in MXene. Additionally, there is a small peak at 57.6° which indicates the presence of V_2_C. Further increase in etching time results in decreased intensity and shifting of the (002) diffraction peak towards a larger angle due to the dissolved V_2_C sheets. The XRD patterns of nanocomposite samples shown in Figure 1b confirm the presence of MnO_2_ along with V_2_CT*_x_*. The broadening of the (002) diffraction peak and the shift towards a lower angle suggests that there is increased interlayer spacing of composite material. The presence of additional broad peaks of the MnO_2_-V_2_CT*_x_* nanocomposite material at a 2Ө value of 35.5° and 39.6° are ascribed to the (112) and (101) planes of polycrystalline orthorhombic MnO_2_ (JCPD 00-0300820) [40]. This broadening of peaks is a result of reduced crystallinity of MnO_2_ over V_2_CT*_x_* sheets. A small peak of V_2_O_5_ near a 2Ө value of 42.7° can be observed, which is produced as a result of heat produced during the etching process [41]. Vanishing of the (002) diffraction peak with the increased weight percent of MnO_2_ is a result of decreased crystallinity of the MnO_2_-V_2_CT*_x_* nanocomposites.

The bandgaps of pristine V_2_C and MnO_2_-V_2_C samples in Figure 1c depict that, similar to pristine MXene, the MnO_2_-V_2_C nanocomposite has shown a direct optical bandgap. The values of energy gained after calculations reveal that pristine V_2_C had a bandgap of 2.22 eV and was reduced to 1.67 eV after the formation of the nanocomposite. The decrease in bandgap is observed upon intercalation of MnO_2_ due to the Mn bonding with the termination sites of V_2_C. The MXene in general are covered with the termination sites that may include -H -O, -F, -OH and -OF [42]. Mn bond with the termination sites of V_2_C thus lead to the creation of defects in the layered MXene. Figure 1d shows the elemental analysis of the MnO_2_-V_2_C nanocomposite. Furthermore, the images obtained from the scanning electron microscope (SEM) in Figure 1e show the layered structure of V_2_C obtained after HF treatment of MAX phase resulting in fanning out and spreading of basal planes, which is a clear indication of a successful etching process. The layered structure in Figure 1f persists even in 10% MnO_2_-V_2_C sample which signifies that the structure of V_2_C has not been destroyed during the formation of the nanocomposite. Figure 1g reveals the TEM image of V_2_C at 200 nm which, when further magnified, clearly shows the layered structure of pristine MXene as in Figure 1h. Moreover, the presence of a whitish carbon layer is observed in Figure 1i, whereas the dark patch is clearly seen over V_2_C sheets in Figure 1i, indicating the presence of MnO_2_; Tang et al. report MnO_2_-Ti_3_C_2_ nanocomposite which reveals a dark patchy structure formed by MnO_2_ over Ti_3_C_2_ sheets [39], similar to that of the MnO_2_-V_2_C nanocomposite.

**Figure 2:**
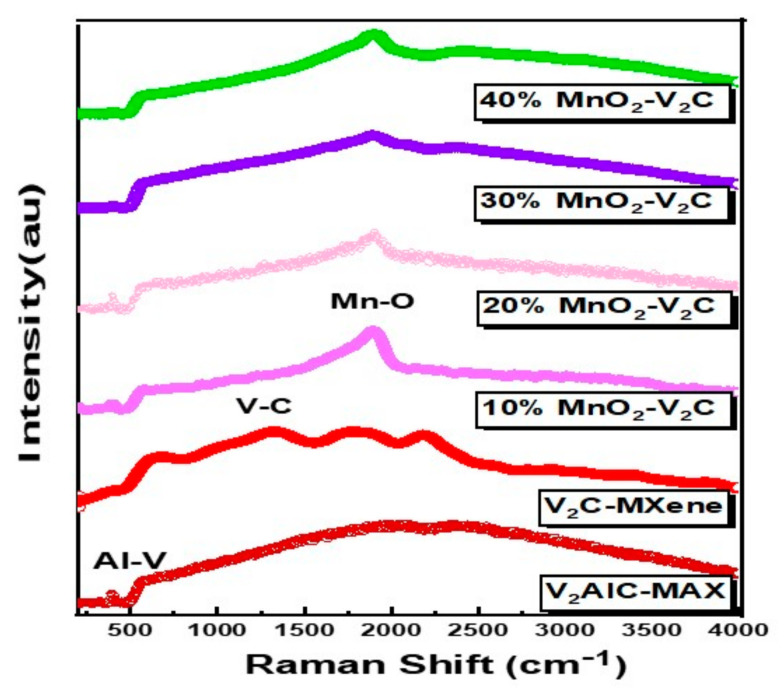
Raman spectroscopy for V_2_AlC, pristine V_2_C and MnO_2_-V_2_C nanocomposite at varying percentages.

In Figure 2, a peak is observed at 394 cm^−1^, which is the characteristic peak for Al-V vibrations [43], that confirms the presence of V_2_AlC, whereas several broad peaks arise in the MXene phase at 657 cm^−1^, 1339 cm^−1^, 1704 cm^−1^, and 2143 cm^−1^ which contribute to V-C vibrations [44]. Along with the peaks of V-C, vibrational peaks of Al-V have also been observed in the nanocomposite samples. This occurs because of the presence of a little amount of MAX phase even after etching. The intensity of modes was enhanced in the MnO_2_-V_2_C nanocomposite when MnO_2_ percentage is increased, compared to pristine V_2_C. In 10% MnO_2_-V_2_C nanocomposite, the peak is much broader compared to 20%, 30% and 40%. This might occur due to the enhancement of motions of atoms after the formation of the nanocomposite. The peaks of MnO_2_ arise around 200 cm^−1^ and 500–600 cm^−1^ [45] while for pristine V_2_C, the peaks arise around 600–2100 cm^−1^. As previously discussed regarding graphene as a parental family to MXenes, Chen et al. reports an MnO_2_/Graphene-Oxide nanocomposite peak around 1750 cm^−1^ [46], which is close enough to vary from the percentage of the MnO_2_-V_2_C nanocomposite peak that is around 1767 cm^−1^. Moreover, for the pristine V_2_C optical modes, the foremost three optical branches in phonon spectrum show considerably lesser frequencies and are near to three acoustic phonon branches, which results in a phonon gap among the lower three and upper three optical branches. This is one of the typical properties of MXene, which is observed in numerous MXene families [47]. Furthermore, substantial contribution comes from vibrations of V-atoms. It can be observed that the motion of V-atoms is weakened by vibrations of the terminal atoms, which concludes a noticeable difference between pristine V_2_C system and V_2_CT*_x_*. Since no robust signal of vanadium oxide was detected in MnO_2_-V_2_C samples, this shows that either the V_2_CT*_x_* sheets are not oxidized, or limited MXene flakes are oxidized beyond the detection of Raman technique, indicating the low density of vanadium oxide on the surface of V_2_CT*_x_*. The remaining peaks shown in the varying percentage samples are of Al-V vibrations, as a little amount of aluminum persists in the pristine MXene sample. Moreover, Figure S1 in Supplementary Materials discussed the FTIR graphs for pristine V_2_C MXene and (10%, 20%, 30% 40%, 50% weight) MnO_2_-V_2_C nanocomposites. The peaks fairly signify the MXene formation and presence of MnO_2_ peaks in MXene plots.

Computational Framework: The computational analysis was carried out with the help of the ab initio all-electron FLAPW method, as executed in the WIEN2k code [33,34]. The calculations were performed using Perdew–Burke–Ernzerhof (PBE) generalized gradient approximation (GGA) exchange-correlation functional [35,48,49]. Ground state structure for V_2_C MXene was attained by relaxation of internal coordinates. Furthermore, the density of states (DOS), band structure, and electronic density was calculated for the relaxed structure using GGA. For a clear description of the experimental observations and to the point information about the structure, the c-lattice parameter was increased up to 22.6 Å. Consequently, the V_2_C MXene nanocomposite relaxed structure had to be generated, for which we used GGA-PBE with 64 k-points in the irreducible Brillouin zone (IBZ). DOS along with the band structure was calculated and analyzed for doped and adsorbed Mn atom on MXene. Wave function in the interstitial regions was expanded in plane waves, with the plane wave cut-off chosen as R_MT_K_max_ = 7.0. R_MT_ represents the smallest radius of the atomic sphere and K_max_ as the largest wave-vector magnitude. The R_MT_ were taken as 1.86 a.u. for V-atoms, 1.55 a.u. for O-atoms, 1.68 a.u. for F-atoms, 1.63 a.u. for C-atoms and 1.80 a.u. for Mn-atom. The K points for structure relaxation is 2 k-points in irreducible Brillouin zone (IBZ) with k-grid of 2 × 2 × 1. In addition to that, k-points for energy convergence are 54 k points in IBZ with k grid of 6 × 6 × 3. Moreover, the forces relaxation criteria were kept at 10^−4^ Ryd and energy convergence criteria was fixed at 10^−5^ Ryd.

**Figure 3:**
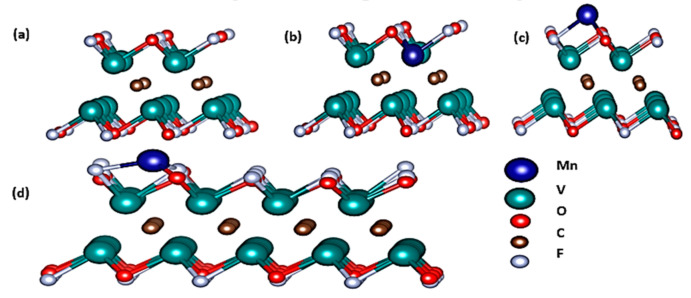
(**a**) Structure of pristine V_2_C, (**b**) structure of Mn-doped V_2_C, (**c**) structure of Mn-adsorbed V_2_C in 2 × 2 × 1 supercell, (**d**) structure of Mn-adsorbed V_2_C in 4 × 4 × 1 supercell.

The system of V_2_C-OF is modelled by a supercell of slabs. Bowman et al. explains the crystal structure of V_2_C [50]. Ideally, simple V_2_C structure could have been considered but herein we inculcated V_2_C along with its surface terminations in order to complement the experimental analysis, because pristine V_2_C cannot exist in ordinary atmospheric conditions. The carbon atom is sandwiched between vanadium layers. The oxygen and fluorine atomic layers were added to the system as functional terminations on to the surface, as shown in Figure 3a. A supercell of 2 × 2 × 1 was initially constructed to examine the stability of manganese in slab using different positions of the Mn atom. For obtaining a stable position of the Mn atom in the V_2_C-OF system, we constructed two different configurations that are by adsorption of the Mn atom and by doping of the Mn atom in the V_2_C-OF system, as shown in Figure 3b,c respectively. The electronic structure of Mn-adsorbed atom was then studied using 4 × 4 × 1 supercell as in Figure 3d.

**Figure 4:**
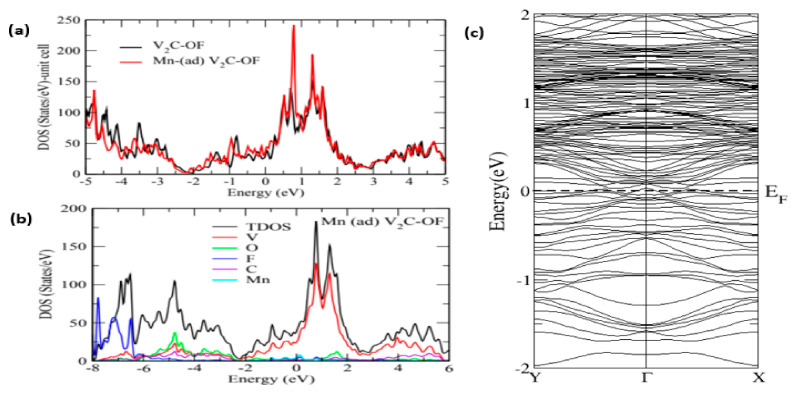
(**a**) DOS vs. energy V_2_C-OF and Mn-adsorbed V_2_C-OF, (**b**) total DOS vs. energy V_2_C-OF and Mn-adsorbed V_2_C-OF, (**c**) electronic bandgap structure for Mn-adsorbed V_2_C-OF.

The electronic structure of the Mn-adsorbed atom was then studied using 4 × 4 × 1 supercell. In the Mn-adsorbed structure, the Mn atom forms a bond with the termination sites, i.e., O and F, while in a doped structure, the Mn-atom substitutes the V-atom. Figure 4a illustrates the density of states (DOS) vs. energy plots for V_2_C-OF and Mn-adsorbed V_2_C-OF. Figure 4a clearly shows that the density of states has been drastically increased in the conduction band around 0 to 2 eV for Mn-adsorbed V_2_C-OF structure as compared to a simple V_2_C-OF system. The increase in major peaks of V in the conduction band is because of the presence of Mn-adsorption sites which are nearer to the V-atomic sites.

From Figure 4b, it is seen that Mn peaks are existing in the conduction band around 0–1 eV and are contributing to the total density of states (TDOS) which is the reason for an increase in the density of states. Moreover, Figure 4c shows the bandgap structure for Mn-adsorbed V_2_C-OF that shows complete metallic behavior, since the bandgap theoretically obtained for pristine MXene is zero [51,52,53]. The experimentally obtained bandgap, however, is found to be different from the one theoretically explained. Since the experimentally obtained data of the bandgap structure has shown a decrease in bandgap, it thus concludes the enhancement in the electronic properties of V_2_C structure.

**Figure 5:**
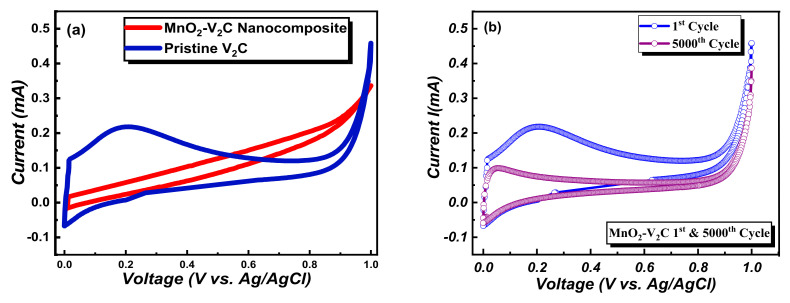

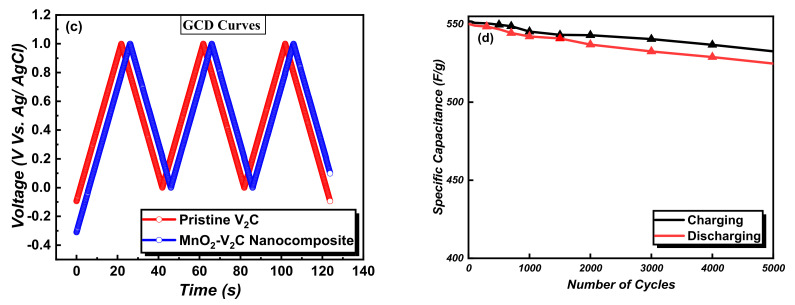
(**a**) Current vs. voltage of pristine MXene and MnO_2_-V_2_C nanocomposite, (**b**) comparison of 1st and 5000th cycle of MnO_2_-V_2_C nanocomposite, (**c**) galvanostatic charge–discharge curves of V_2_C and MnO_2_-V_2_C, (**d**) specific capacitance vs. number of cycles.

Electrochemical Analysis: The cyclic voltammetry (CV) reveals the I-V curves of pristine V_2_C and the MnO_2_-V_2_C nanocomposite, which was discerned at a potential window of 0.0 V to +0.9 V while the scan rate was fixed as 100 mV/s. V_2_C however oxidizes rapidly due to its surface reactivity [54,55] but the oxidized samples can be investigated by storing diluted samples of V_2_C in sealed Eppendorf vials at room temperature and analyzing the samples first. Furthermore, MnO_2_ adsorption in V_2_C helps in omitting unwanted surface attachments. Figure 5a shows the current vs. voltage graph, which reveals that the gravimetric capacitance for V_2_C is about 196.5 F/g, larger than the value reported in [29], and the highest gravimetric capacitance obtained for the MnO_2_-V_2_C nanocomposite is about 551.8 F/g. The enhanced value of gravimetric capacitance of the MnO_2_-V_2_C nanocomposite is more than twice that obtained for pristine MXene.

MnO_2_-V_2_C nanocomposite exhibited high values of specific capacitance compared to pristine V_2_C. It is encompassing all other factors, i.e., a high specific area and increased storage ability, due to its morphology which is generally a flake-like structure. At comparably higher current density, K^+^ ions are diffused from the 1 M KOH electrolyte into the nanocomposite and gain access i.e., they penetrate into the gaps available between nanocomposite layers easily, which leads to an efficient charge–discharge ratio [56]. Until 5000 cycles, the results are excellent as the 5000th cycle’s gravimetric capacitance obtained was 532.6 F/g. The reason for decreased efficiency is the degradation of electrode material, though it signifies that retentivity is very high, at about 96.5% of the original value. Figure 5b shows a comparison of the nanocomposite’s 1st cycle and 5000th cycle curve, which reveals that theoretical study has complemented the experimental data. The theoretical results stated that with the enhancement of electronic density and the stability of adsorbed Mn-atom in MXene, which is fairly seen by the comparison graph of current-voltage cycles, a high specific capacitance has been achieved after Mn adsorption with viable stability up to 5000 cycles. Bare V_2_C cannot show such a high specific capacitance due to hydrophilicity and thus readily oxidizes in the atmosphere. Figure 5d adds to the argument of cyclic stability of the nanocomposite, which also reveals that a very high retentivity percentage of specific capacitance has been achieved, even after 5000 cycles. Moreover, the obtained galvanostatic charge–discharge triangular curves for pristine V_2_C and MnO_2_-V_2_C can be seen in Figure 5c, revealing an outstanding and improved performance of the nanocomposite electrode material, as it is providing a high gravimetric capacitance even after a longer time period.

## 4. Conclusions

The two-dimensional materials with the general formula V_2_CT*_x_* were synthesized after wet-chemical etching from the bulk parent compound MAX. This article reported experimental as well as theoretical outcomes on structural, morphological and optoelectronic properties of pristine MXene and MnO_2_-MXene nanocomposites. XRD revealed that the c-lattice parameter increased from 13.01 Å to 22.6 Å for MAX and MXene respectively, and then reached 27.2 Å for the MnO_2_-V_2_C nanocomposite, signifying adsorption-dominant properties. SEM, EDX, and bandgap analysis demonstrated that the adsorption of MnO_2_ in V_2_C, which showed an intriguing gravimetric capacitance in the MnO_2_-MXene nanocomposite of approximately 551.8 F/g, has a retentivity of about 96.5% after 5000 cycles. The computational analysis supported the experimental data as the density of states inevitably increased when MnO_2_ was adsorbed in V_2_C.

## Data Availability

The data supporting the findings of “Experimental and Computational Analysis of MnO_2_@V_2_C-MXene for Enhanced Energy Storage” are available within the manuscript and the corresponding supporting information file.

## Supplementary Materials

The following are available online at https://www.mdpi.com/article/10.3390/nano11071707/s1, Figure S1 in Supplementary Materials discussed the FTIR graphs for pristine V_2_C MXene and (10%, 20%, 30% 40%, 50% weight) MnO_2_-V_2_C nanocomposites. The peaks fairly signify the MXene formation and presence of MnO_2_ peaks in MXene plots.
