# Supplementary material for: Experimental and Computational Analysis of MnO2@V2C-MXene for Enhanced Energy Storage
Source: Nanomaterials (Basel). 2021 Jun 29;11(7):1707. doi: 10.3390/nano11071707 (PMC8308169; doi:10.3390/nano11071707)
Supplement: Supplementary file 1 [file nanomaterials-11-01707-s001.zip › nanomaterials-1179431-supplementary.pdf]

Supplementary Materials:

Experimental and Computational Analysis of MnO_2@V_2C-MXene for Enhanced Energy Storage

Figure S1 show FTIR plots for pristine V_2C MXene and (10%, 20%, 30% 40%, 50% weight) MnO_2/V_2C nanocomposites. The peak at 2337 cm^{-1} is typically seen for the MXene sheets [1]. An intense peak is also observed at 2074cm^{-1} represent to stretching of C ≡ C bond while a small bump is also observed around 1975cm^{-1} which contributes to C=C and C=O vibrations [2]. The peaks in the region between 500 cm^{-1} to 550 cm^{-1} are the characteristic peak of V-O bands [3]. A peak is majorly observed in MnO_2-V_2C nanocomposite samples around 936-1031cm^{-1}. This peak does not exist in Pristine MXene and is only existing in MnO_2-V_2C composite which is an evidence contributing to Mn=O bonds presence and shows the presence of MnO_2 with V_2C as M Mylarappa et al. shows transmittance peaks of MnO_2 [4]. The layered structure of V_2C samples exists after MnO_2 adsorbed at variated percentage intercalation in V_2C till 40% however, the destruction caused in V_2C sheets is comparably higher when we increase MnO_2 percentage beyond 40% in the structure with the destruction of MXene sheets. At equal ratios, i.e. 50% of MnO_2-V_2C ratio, the V=C bonds are not observable and different peaks at 1601cm^{-1} and 1343cm^{-1} arise which contribute to the presence of Mn=O bonds [5]. Several small transmission peaks around 1000–1500cm^{-1} are assigned to bending vibrations of bonds of O–H which are connected with Mn-atoms [6].

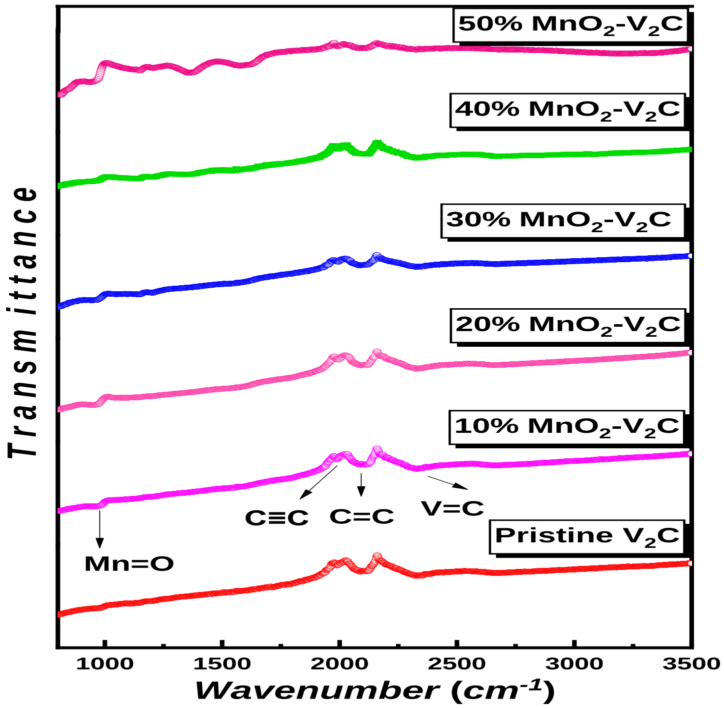

Figure S1. FTIR plots for Pristine MXene V_2C and MnO_2-V_2C nanocomposite at varying percentages.

Raman Spectroscopy was initiated at room temperature for analysis of the acoustic and optical modes. Argon ion laser radiation wavelength 514 nm was focused to a spot size of ~1 μm with an incident power kept below 1mW. The knowledge about phonon spectrum of V_2AlC, Pristine V_2C and MnO_2@V_2C composite can be obtained which has several broad peaks ranging from 200 to 2100 cm^{-1} and is assigned as non-stoichiometric. The crystallographic structure of V_2C has 3 atoms of the primitive unit cell in V_2C monosheet which give rise to 6 optical modes and 3 acoustic modes at the Γ point of the Brillouin

zone [7]. The information regarding the phonon spectrum is essential for understanding macroscopic properties such as electrical and thermal properties, so the phonon spectrum of Pristine V_2C was studied from literature, also Gao et al. reports the phonon dispersions for V_2C [8]. Without terminations, V_2C consist of three acoustic modes from which two acoustic modes exhibit linear dispersion near Γ and thus correspond to an in-plane vibrations. The G band is a result of in-plane vibrations of sp^2 bonded Carbon atoms. On the contrary, third acoustic mode corresponds to an out-of-plane vibration of V-atoms and has a quadratic dispersion close to Γ. This out of plane vibration of V-atoms results in D band and is attributed to the presence of structural defects. The above-mentioned quadratic dependence has an analogy to the one observed in graphene, which is also considered as parental family to MXenes system [9]. V_2C with termination groups, such as -F and -OH results in different optical and acoustic modes as in the case of V_2CF_2, the 5 atoms of primitive unit cell led to 12 optical modes and 3 acoustic modes at the Γ point of the Brillouin zone. In the case of $V_2C(OH)_2$, the 7 atoms of each primitive cell led to 18 optical modes and 3 acoustic modes at the Γ point of the Brillouin zone.

References

1. Rafiq S, Awan S, Zheng R, Wen Z, Rani M, Akinwande D and Rizwan S. Novel room-temperature ferromagnetism in Gd-doped 2-dimensional Ti_3C_2 T x MXene semiconductor for spintronics. *J. of Magnetism and Magnetic Materials.* **2019**,165954.
2. Luo J, Tao X, Zhang J, Xia Y, Huang H, Zhang L, Gan Y, Liang C and Zhang W. Sn^{4+} Ion Decorated Highly Conductive Ti_3C_2 MXene: Promising Lithium-Ion Anodes with Enhanced Volumetric Capacity and Cyclic Performance. *ACS Nano*. **2016**, *10*, 2491–2499, doi:10.1021/acsnano.5b07333.
3. Haining Ji, Dongqing Liu, Haifeng Cheng, Chaoyang Zhang, Lixiang Yang and Dewei Ren. Infrared thermochromic properties of monoclinic VO_2 nanopowders using a malic acid-assisted hydrothermal method for adaptive camouflage. *RSC Adv*., **2017**, *7*, 5189–5194.
4. M Mylarappa, V Venkata Lakshmi, K R Vishnu Mahesh, H P Nagaswarupa and N Raghavendra. A facile hydrothermal recovery of nano sealed MnO_2 particle from waste batteries: An advanced material for electrochemical and environmental applications. *IOP Conf. Ser.: Mater. Sci. Eng.* **2016**, *149*, 012178.
5. M. Minakshi, P. Singh, T.B. Issa, S. Thurgate, R.D. Marco, J. Power Sources. Lithium insertion into manganese dioxide electrode in MnO_2/Zn aqueous battery Part II. Comparison of the behavior of EMD and battery grade MnO_2 in Zn|MnO_2| aqueous LiOH electrolyte. *J. of Power Sources*. **2004**, *138*, 319–322.
6. D.P. Dubal, W.B.Kim, C.D.Lokhande. Galvanostatically deposited Fe: MnO_2 electrodes for supercapacitor application. *J. of Physics and Chemistry of Solids*. **2012**, *73*, 18–24.
7 Aur_elie Champagne, Lu Shi, Thierry Ouisse, Benoît Hackens, and Jean-Christophe Charlier. Electronic and vibrational properties of V_2C-based MXenes: From experiments to first-principles modelling. *Phys. Rev*. **2018**, *B 97*, 115439.
8. Guoying Gao, Guangqian Ding, Jie Li, Kailun Yao, Menghao Wu and Meichun Qian. Monolayer MXenes: Promising half-metals and spin gapless semiconductors. *Nanoscale*. **2016**, *8*, 8986–8994.
9. M. S. Dresselhaus and P. C. Eklund. Phonons in carbon nanotubes. *Adv. Phys*. **2000**, *49*, 705.
